# E-Cigarette Narratives of User-Generated Posts on Xiaohongshu in China: Content Analysis

**DOI:** 10.2196/71173

**Published:** 2025-07-03

**Authors:** Tingfen Ji, Zhao Liu, Zheng Su, Xin Xia, Yi Liu, Ying Xie, Zhenxiao Huang, Xinmei Zhou, Min Wang, Anqi Cheng, Qingqing Song, Yuxin Shi, Shunyi Shi, Aihemaiti Ailifeire, Jiahui He, Yingman Gao, Liang Zhao, Liyan Wu, Dan Xiao, Chen Wang

**Affiliations:** 1 China-Japan Friendship School of Clinical Medicine, Capital Medical University, Beijing, China; 2 Department of Tobacco Control and Prevention of Respiratory Diseases, Center of Respiratory Medicine, China-Japan Friendship Hospital, Beijing, China, 86 010 8420 5288, 86 010-64217749; 3 WHO Collaborating Center for Tobacco Cessation and Respiratory Diseases Prevention, No. 2, Cherry Blossom East Street, Chaoyang District, Beijing, China; 4 National Clinical Research Center for Respiratory Diseases, Beijing, China; 5 National Center for Respiratory Medicine, Beijing, China; 6 Peking Union Medical College, Chinese Academy of Medical Sciences, Beijing, China; 7 Central Sterile Supply Department, WangJing Hospital of China Academy of Chinese Medical Sciences, Beijing, China

**Keywords:** social media, qualitative research, vaping, RedNote, teenagers

## Abstract

**Background:**

Social media platforms have become influential spaces for disseminating information about electronic cigarettes (e-cigarettes). Concerns persist about the spread of misleading content, particularly among social media vulnerable groups. Xiaohongshu (RedNote), widely used by Chinese youth, plays a growing role in shaping e-cigarette perceptions. Understanding the narratives circulating on this platform is essential for identifying misinformation, assessing public perception, and guiding future health communication strategies.

**Objective:**

This study aimed to analyze the content, topics, user engagement, and sentiment trends of e-cigarette–related posts on Xiaohongshu and to assess the factors that influence engagement.

**Methods:**

E-cigarette–related posts published on Xiaohongshu between January 2020 and November 2024 were collected using web scraping, based on a predefined keyword list and a time-stratified random sampling strategy. Posts were categorized into 4 themes: advertising promotion, health hazards, usage interaction, and others. High-frequency keywords were extracted, and representative quotes were included to illustrate user perspectives across each category. Sentiment analysis was performed on posts in the usage interaction category to assess public attitudes. We defined 4 sentiment categories: positive, negative, neutral, and mixed. Logistic regression was conducted to explore the effects of post type, content length, and thematic classification on user engagement metrics such as likes, saves, and comments.

**Results:**

A total of 1729 posts were included and analyzed. Usage interaction posts were the most common (681/1729, 39.39%), with keywords such as “experience,” “regulations,” and “quit smoking” dominating this category. Advertising promotion posts (512/1729, 29.61%) frequently used terms like “flavor,” “fashion,” and “design” to attract younger users. Health hazards posts (311/1729, 17.99%) highlighted risks with keywords like “nicotine,” “addiction,” and “secondhand smoke,” while others included policy and industry updates. Representative quotes highlighted typical concerns about aesthetics, health risks, and cessation struggles. Health hazards posts garnered the highest engagement in terms of likes and saves, despite their limited presence (odds ratio [OR] 1.498, 95% CI 1.099‐2.042, *P*=.01). Video posts significantly outperformed text-image posts in generating comments (OR 2.624, 95% CI 2.017‐3.439, *P*<.001). Sentiment analysis of the usage interaction posts (n=681) revealed that 53.45% (364/681) were positive, highlighting reduced harm, convenience, or flavor preferences. Negative sentiment was observed in 33.48% (228/681) of posts, often expressing concerns about addiction and health risks. Mixed sentiments appeared in 6.90% (47/681), acknowledging both pros and cons. In addition, 6.17% (42/681) of posts were classified as neutral without evident emotional tone.

**Conclusions:**

The findings underscore the dual role of Xiaohongshu as a platform for both e-cigarette promotion and public discourse. Misleading marketing targeting vulnerable groups, such as adolescents, remains a critical issue. However, the strong user response to health-related content suggests that social media platforms could be leveraged for effective health education. Strengthened regulatory oversight and educational campaigns leveraging engaging content formats are urgently needed to counter misinformation and protect public health.

## Introduction

The rapid expansion of social media platforms has reshaped how individuals access, share, and perceive information [1]. Among these platforms, Xiaohongshu (also known as RedNote), one of China’s leading social media apps, stands out for its diverse content ecosystem where users engage in sharing lifestyle-related experiences [2]. Unlike Reddit and Facebook (Meta), which primarily serve as communication platforms, Xiaohongshu uniquely integrates social interaction with e-commerce, creating a seamless experience that allows users to discover, share, and shop [3]. Similar to Instagram (Meta), but with an emphasis on authentic user-generated content, Xiaohongshu’s distinguishing feature is its advanced personalization algorithm, which analyzes user preferences to provide tailored content and product recommendations, greatly improving user engagement and shopping efficiency.

Electronic cigarettes (e-cigarettes) are consumer tobacco products engineered to deliver nicotine through the generation of an aerosol, which contains pharmacologically active substances, flavorings, and other chemical components present in e-liquids [4]. In recent years, e-cigarettes have emerged as a contentious topic on social media due to their dual nature as both lifestyle products and potential health hazards [1,5,6]. This duality makes Xiaohongshu an intriguing case for studying user-generated content related to e-cigarettes. In China, this issue is further complicated by the evolving regulatory environment. National authorities have introduced stricter controls on e-cigarette marketing and access. Online sales and advertising of e-cigarettes have been officially banned since 2019. E-cigarettes were formally included in the tobacco product category for inclusion in 2021, and further regulations introduced in 2022 limit available flavors to tobacco flavors and require product standardization [7,8].

Despite growing global concern over the impacts of e-cigarettes, especially among young users [9], research on how such products are portrayed and discussed on social media platforms remains limited [10]. Timely insights into social media trends can enhance our understanding of e-cigarette product dynamics, which significantly affect consumer behavior [11]. Previous studies have demonstrated that social media can significantly influence e-cigarette use among users [12]. However, these studies primarily analyze social media data from the United States and European countries [13-15]. Understanding how e-cigarettes are discussed on a popular platform like Xiaohongshu in China provides valuable insights into public perceptions and potential health communication strategies. Given the platform’s extensive reach among young consumers, the findings of this research could inform future public health campaigns and regulatory policies aimed at mitigating the adverse health effects of e-cigarette use.

Therefore, this study aimed to examine user-generated content related to e-cigarettes on Xiaohongshu, with a particular focus on thematic patterns, sentiment distribution, and factors influencing user engagement. Specifically, we analyzed posts published between 2020 and 2024 to explore how e-cigarettes are portrayed, emotionally framed, and interacted with on a platform widely used by young consumers in China.

## Methods

### Overview

**Figure 1.**
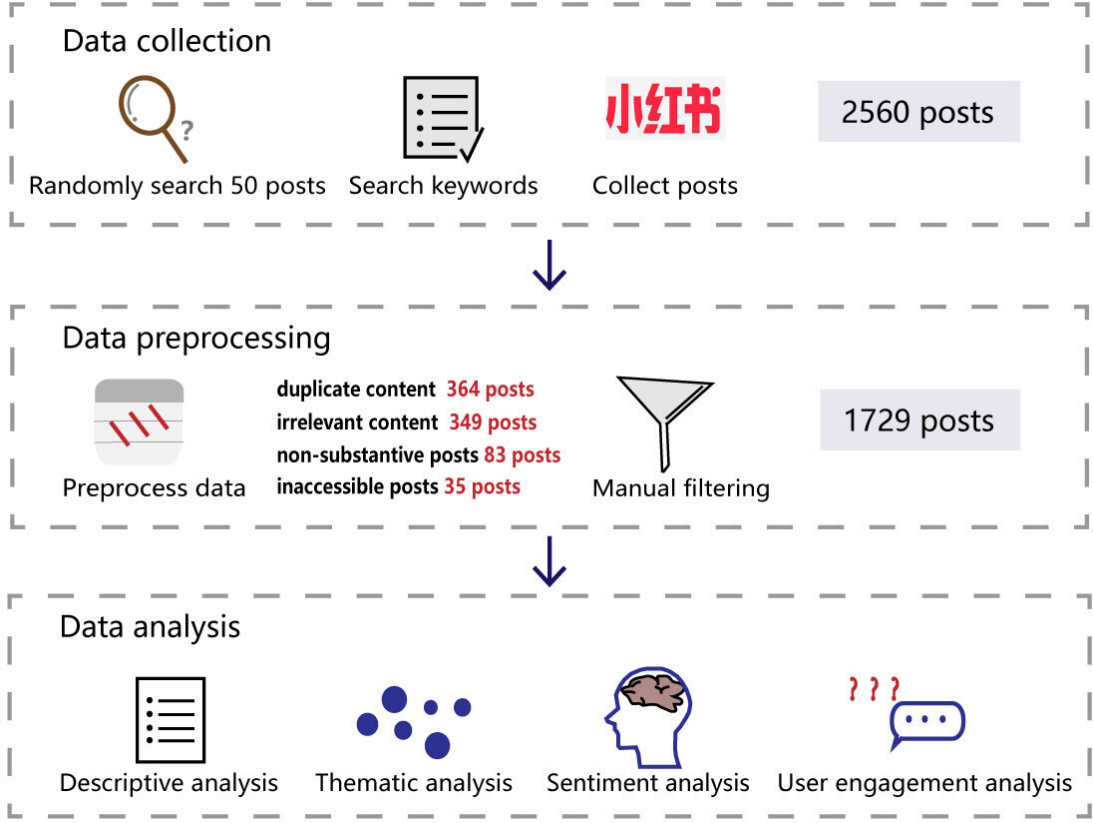
Flowchart of the study.

The study process consisted of several steps to prepare, collect, clean, and analyze data on e-cigarette–related posts from Xiaohongshu (Figure 1).

### Keyword List and Web Scraping

Two researchers (TJ and ZL) randomly searched 50 posts containing e-cigarette from Xiaohongshu as an initial sample to establish a keyword list (Textbox 1). Due to the large volume of platform content and limited data access, we adopted a time-stratified random sampling strategy to ensure broad temporal coverage. The observation period (January 2020 to November 2024) was divided into monthly intervals. For each month, one calendar day was randomly selected using the “slice_sample” function in R (R Core Team). On each sampled date, the research team retrieved publicly accessible posts based on keyword lists. Drawing on the work of Bao et al [16-18], we implemented a web crawling pipeline using Python 3.11 and MediaCrawler, incorporating the urllib library for web requests and content parsing [19]. No ranking or filtering by engagement or popularity was applied during retrieval.

Textbox 1. **Search keywords on Xiaohongshu.**

**Search keywords**

- 电子烟
- 電子煙
- 电 zi 烟
- 电子 yan
- 悦刻
- 悦客
- Relx
- 悦 KE
- Iqos
- 悦科
- Glo
- 电子 y

### Data Cleaning

The collected dataset of posts underwent a rigorous data cleaning process to enhance quality and relevance for subsequent analysis. Initially, 2 researchers (TJ and ZL) independently screened all posts to exclude those unrelated to e-cigarettes. Any disagreements between the 2 researchers were resolved by a third researcher (ZS) to ensure consistency.

Manual filtering excluded posts based on the following criteria: (1) duplicate content—posts that were identical or highly similar to previously collected entries; (2) irrelevant content—posts that contained keywords such as “electronic” or “smoke” but were unrelated to e-cigarettes (eg, referring to electronic devices or photography effects); (3) nonsubstantive posts—those containing only emojis, promotional codes, broken links, or extremely limited text (eg, 1‐2 words); and (4) inaccessible posts—those that could not be retrieved due to deleted user accounts, broken URLs, or platform restrictions.

For all retained posts, essential metadata such as URLs, publishers, publication dates, and user engagement metrics was extracted. Text data from video-based posts were also included to maximize the comprehensiveness of the dataset.

The text content of each post was subjected to preprocessing to remove irrelevant elements such as numbers, spaces, emoticons, hyperlinks, punctuation marks, and filler words. Common stopwords were also eliminated to focus on meaningful textual components.

### Descriptive Analysis

Descriptive statistics were calculated to summarize the characteristics of e-cigarette–related posts. These included post type (eg, image-text or video), content length (eg, long posts or short posts), and user engagement metrics.

### Thematic Analysis

Thematic analysis followed Braun and Clarke’s framework for thematic analysis [20]. The research team familiarized themselves with the data by carefully reading the full content of each post. Then, the coding was conducted using the qualitative data analysis software Nvivo. Posts were categorized into four classifications: advertising promotion, health hazards, usage interaction, and others.

Each classification is subdivided into subtopics based on different aspects of e-cigarette discourse on Xiaohongshu. Advertising promotion included content focusing on product features, branding, and aesthetic appeal; health hazards encompassed discussions on nicotine addiction, health risks, and secondhand smoke exposure; usage interaction covered user experiences, smoking cessation attempts, and social perceptions of e-cigarette use; and Others included industry updates, policy discussions, and recruitment information.

To enhance reliability, 2 researchers independently coded the dataset. Discrepancies in coding were resolved through discussion, and consensus was achieved on the final themes. Saturation was reached when no new themes emerged during the analysis process. The team also conducted word segmentation using Jieba 0.42 to identify and quantify frequently used terms in each theme. In addition to keyword extraction, we incorporated representative quotes for each subtopic to better capture the context and expressive content of posts. These quotes were used to illustrate how specific themes were articulated by users and to support the interpretation of nuanced trends.

### Sentiment Analysis

Sentiment analysis was conducted to assess the emotional tone of posts within the usage interaction theme, focusing on user attitudes toward e-cigarettes.

Each post was categorized as positive, negative, neutral, or mixed sentiment based on a structured manual annotation protocol [1]. Two independent coders applied predefined criteria to label posts, and a third researcher adjudicated any discrepancies to ensure consistency. Positive posts were defined as those expressing approval or acceptance of e-cigarettes, often highlighting perceived benefits. Negative posts conveyed disapproval or concern, typically citing health risks, addiction, or dissatisfaction. Neutral posts lacked evident emotional bias and contained descriptive or objective content. Mixed sentiment referred to posts that acknowledged both positive and negative aspects of e-cigarette use, such as perceived benefits in convenience or stress relief alongside concerns about health risks or addiction.

We assigned sentiment scores using the number of likes each post received. The like counts were log-transformed [score=log10(likes +1)] to reduce skewness and mitigate the impact of outliers. Posts with positive sentiment were assigned a positive score equal to their log-transformed like count, and negative sentiment posts were assigned a negative score (ie, the negative of their log-transformed like count). Posts with neutral or mixed sentiment were considered emotionally ambiguous and were therefore assigned a score of zero. A scatter plot was generated to visualize sentiment polarity, with the horizontal axis representing individual posts and the vertical axis denoting sentiment scores.

### User Engagement Analysis

Multivariate logistic regression analysis was conducted to evaluate factors influencing user engagement. The dependent variable was the likelihood of receiving user comments, while independent variables included post type, thematic classification, and content length. Posts were grouped into “high engagement” (≥10 comments) and “low engagement” (<10 comments). To ensure the validity of the logistic regression model, we assessed multicollinearity among independent variables using variance inflation factors. Odds ratios (ORs) with 95% CIs were calculated to determine the strength and significance of associations. A *P* value of <.05 was considered statistically significant.

### Ethical Considerations

All the data in this paper were obtained from Xiaohongshu’s public data, which protects those who have private profiles from being subject to research studies. Furthermore, we desensitized the data to protect the privacy of the users. No personal identifiers (eg, usernames, profile photos, user IDs) were collected or retained. To minimize reidentification risk, representative quotes were paraphrased where necessary. As the data were public, anonymized, and analyzed in aggregate without user interaction, the study was deemed exempt from institutional review board review.

## Results

### Overview of E-Cigarette–Related Posts

As of November 2024, a total of 2560 e-cigarette–related posts were collected, and 1729 posts were included in this analysis after preprocessing. Among 1729 posts, 72.8% (1,258/1729 posts) were image-text posts, while 27.2% (471/1729 posts) were video-based posts. Longer posts (≥100 words) comprised 58.7% (1015/1729 posts), while shorter ones (<100 words) accounted for 41.3% (714/1729 posts). Usage interaction posts accounted for the largest proportion (681/1729, 39.39%), followed by advertising promotion (512/1729, 29.61%), health hazards (311/1729, 17.99%), and others (225/1729, 13.01%). The results were shown in Table 1.

**Table 1.** Basic characteristics of Xiaohongshu posts on e-cigarettes.

Classification	Advertising promotion	Health hazards	Usage interaction	Others
Total number, n (%)	512 (29.61)	311 (17.99)	681 (39.39)	225 (13.01)
Posting year, n (%)				
January 2020‐December 2020	4 (28.57)	2 (14.29)	6 (42.86)	2 (14.29)
January 2021‐December 2021	23 (26.14)	31 (35.23)	27 (30.68)	7 (7.95)
January 2022‐December 2022	19 (21.35)	36 (40.45)	25 (28.09)	9 (10.11)
January 2023‐December 2023	63 (24.23)	58 (22.31)	98 (37.69)	41 (15.77)
January 2024‐December 2024	403 (31.53)	184 (14.40)	525 (41.08)	166 (12.99)
Post type, n (%)				
Text-image posts	361 (28.70)	191 (15.18)	537 (42.69)	169 (13.43)
Video posts	151 (32.06)	120 (25.48)	144 (30.57)	56 (11.89)
Content length, n (%)				
Long posts (≥100 words)	282 (27.78)	217 (21.38)	309 (30.44)	207 (20.39)
Short posts (100 words)	230 (32.21)	94 (13.17)	372 (52.10)	18 (2.52)
Engagement, n (%)				
Likes	33,650 (7.34)	198,059 (43.21)	177,513 (38.73)	49,169 (10.73)
Saves	12,650 (8.31)	62,692 (41.20)	48,519 (31.88)	28,321 (18.61)
Comments	18,169 (15.25)	22,546 (18.92)	66,641 (55.93)	11,801 (9.90)

**Figure 2.**
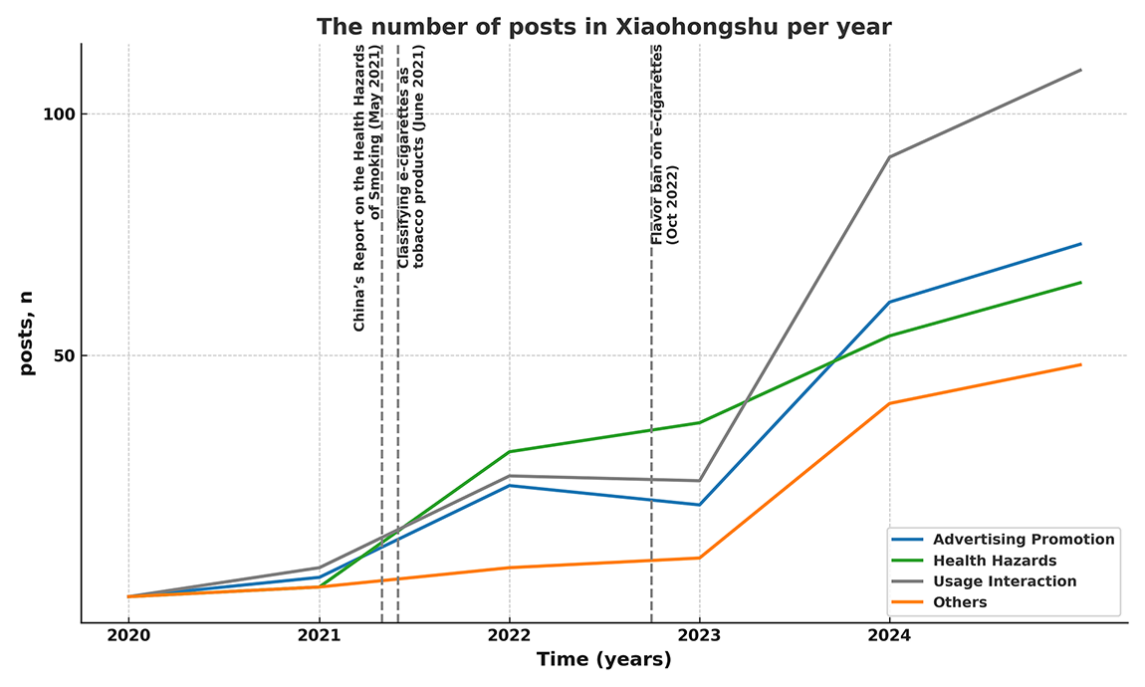
The number of different classification posts in Xiaohongshu and time of significant e-cigarette regulation and report in China (January 2020‐January 2024).

The temporal distribution of posts showed an increasing trend over time. Before December 2020, only 14 posts were recorded (14/1729, 0.81%), while 2021 and 2022 accounted for 88 (88/1729, 5.09%) and 89 (89/1729, 5.15%) posts, respectively. The number of posts surged in 2023, reaching 260 (260/1729, 15.03%) posts (Figure 2).

In terms of engagement, the posts received 458,391 likes, 152,182 saves, and 119,157 comments. Usage interaction posts generated the highest number of comments (66,641/119,157, 55.93%), followed by health hazards (22,546/119,157, 18.92%) and advertising promotion (18,169/119,157, 15.25%).

### Content Themes and Keywords

The advertising promotion category included subtopics such as product features (123/1729, 7.11%), branding (316/1729, 18.27%), and aesthetics (73/1729, 4.22%). Keywords like “flavor,” “design,” “fashion,” and “variety” were frequently used. Much of the content emphasized flavor variety, personalized appearance, and low harm.

The health hazards theme focused on nicotine addiction (94/1729, 5.44%), health impacts (180/1729, 10.41%), and secondhand smoke (37/1729, 2.14%). In addition to common keywords such as “nicotine,” “addiction,” and “harms,” some posts described products disguised as candy or milk tea cups and mentioned secondhand smoke-like effects of e-cigarettes.

The usage interaction theme was subdivided into user experiences (445/1729, 25.74%), smoking cessation attempts (85/1729, 4.92%), and public smoking concerns (151/1729, 8.73%). Posts frequently referenced withdrawal symptoms, flavor recommendations, and the increasing visibility of e-cigarette use among students and in public spaces.

The others category included industry updates (16/1729, 0.93%), policy discussions (152/1729, 8.79%), and recruitment information (57/1729, 3.29%), with keywords such as “regulations,” “prohibition,” and “market”. Recruitment information included posts related to employment opportunities, such as civil service positions at tobacco regulatory agencies or job openings in e-cigarette companies (Table 2).

**Table 2.** Classification and subtopics and keywords of Xiaohongshu posts.

Classification and subtopics	Keywords	Representative quotes	Value, n (%)
Advertising promotion			512 (29.61)
Product features	exhibition, e-liquid, cartridge, quit smoking, gift, atomizer	“Focused on user health, using harmless e-liquid ingredients to reduce harmful substances.” “E-cigarettes may be more effective for quitting smoking—they satisfy the need to puff without much burden.”	123 (7.11)
Branding	RELX, flavor, phantom, experience, fruit, variety	“Let me introduce this lychee-flavored Phantom brand today—it has a strong fruity aroma and feels great!” “Let’s talk about the irresistible flavors of RELX Phantom 5th generation!”	316 (18.27)
Aesthetics	design, usage, product, color, fashion, recommendation	“Every detail is exquisitely crafted—the color combinations and high-quality materials are truly irresistible.” “Its stylish and lightweight design is perfect for girls on the go.”	73 (4.22)
Health hazards			311 (17.99)
Nicotine addiction	nicotine, health, quit smoking, traditional, addiction, ingredients	“Does vaping mean you won’t get addicted?” “Although e-liquid doesn’t contain tar, it usually contains nicotine—a highly addictive substance.”	94 (5.44)
Health impacts	harm, health, hospital, impact, stress, disease	“The harmful gases and particles from e-cigarettes can enter the lungs and may increase the risk of lung cancer, pneumonia, bronchitis, and emphysema.” “That cute milk tea cup sold at the school gate? Turns out it’s actually a vape—watch this video to learn the risks.”	180 (10.41)
Secondhand smoke	secondhand smoke, vapor, impact, harm, ingredients, tar	“Ever catch a whiff of something sweet on the street and think it’s a dessert shop—only to turn around and see someone blowing clouds?” “E-cigarettes can still pose secondhand smoke risks, though more subtly.”	37 (2.14)
Usage interaction			681 (39.39)
User experiences	experience, flavor, like, recommendation, mint, friend	“The apple flavor is really nice—equivalent to about 7 pods, with just the right sweetness and a cool finish.” “I’m bored with the usual pods—any flavor recommendations?”	445 (25.74)
Smoking cessation attempts	attempt, cigarette, withdrawal symptoms, success, stress, health	“It’s really hard—I crave it at work, in the restroom, basically all the time.” “Why is quitting e-cigarettes even harder than quitting traditional ones?”	85 (4.92)
Social perceptions of e-cigarette use	public places, regulations, impact, environment, culture, teenagers	“Not sure when it started, but e-cigarettes are being sold everywhere now—and more and more young people are using them. I even noticed lots of students carrying those little tubes.” “A man vaping in the hallway of a maternity and child health hospital sparked controversy.”	151 (8.73)
Others			225 (13.01)
Industry updates	market, product, brand, report, US, customer	“If traditional cigarette market share keeps falling, US may switch to e-cigarettes or nicotine pouches within three years.” “Top 10 global platforms to know for new vape product releases.”	16 (0.93)
Policy discussions	regulations, prohibition, environment, safety, country, sale	“In foreign countries, RELX and other e-cig brands are subject to strict legal oversight—all products must be approved before being sold.” “E-cigarettes are banned in Macau—violators face fines.”	152 (8.79)
Recruitment information	interview, position, company, job, problem, profession	“Shaanxi Tobacco just posted a recruitment notice—82 positions available, online registration only.” “We’re a vape export company urgently hiring international sales reps for frequent short-term travel.”	57 (3.29)

**Figure 3.**
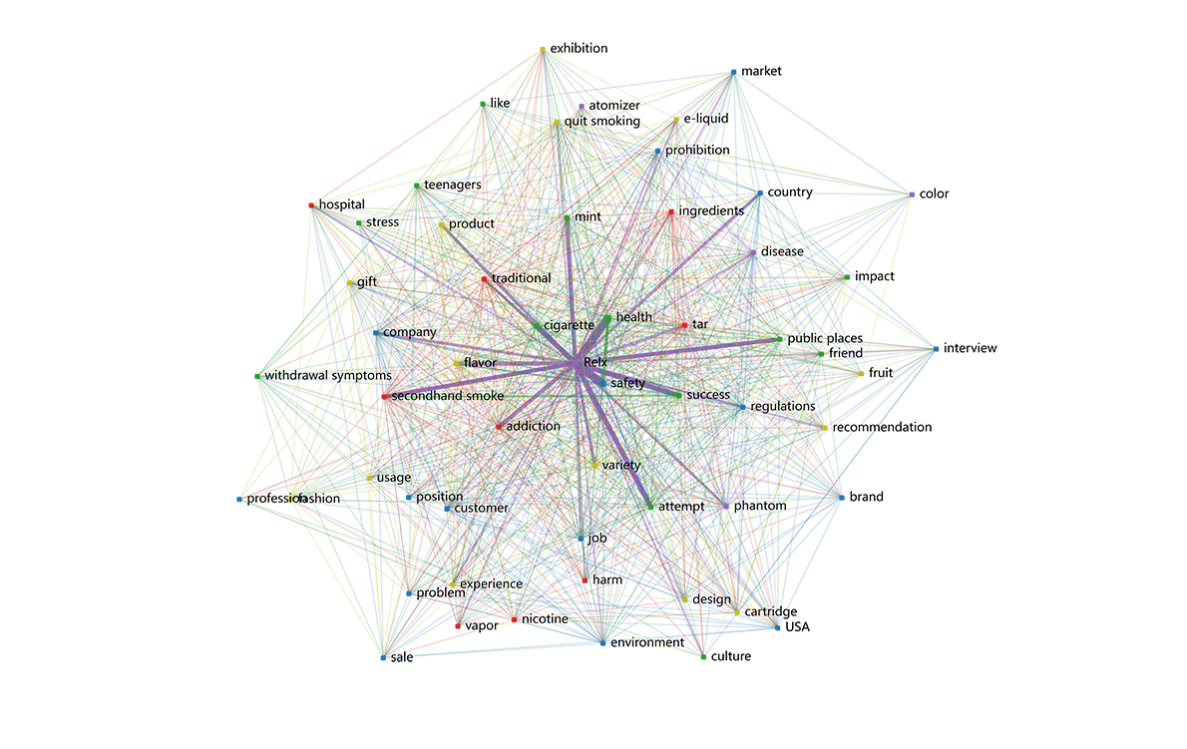
Co-occurrence network of high-frequency keywords of 4 classifications on Xiaohongshu.

The co-occurrence network (Figure 3) visualizes the relationships between keywords extracted from the 1729 e-cigarette–related posts. Prominent keywords, including “Relx,” “nicotine,” “health,” “addiction,” “flavor,” and “cigarette,” emerged as central nodes. In the advertising promotion theme, keywords such as “Relx,” “flavor,” “design,” and “fashion” demonstrated dense connections. The health hazards theme exhibited strong connections between keywords such as “nicotine,” “health,” “addiction,” and “secondhand smoke.” For the usage interaction theme, keywords such as “experience,” “attempt,” “success,” and “regulations” formed a distinct cluster.

The network also highlighted overlaps between themes. For instance, “quit smoking” linked the health hazards and usage interaction themes. Similarly, commercial terms like “Relx” bridged the advertising promotion and usage interaction themes.

### Sentiment Analysis

**Figure 4.**
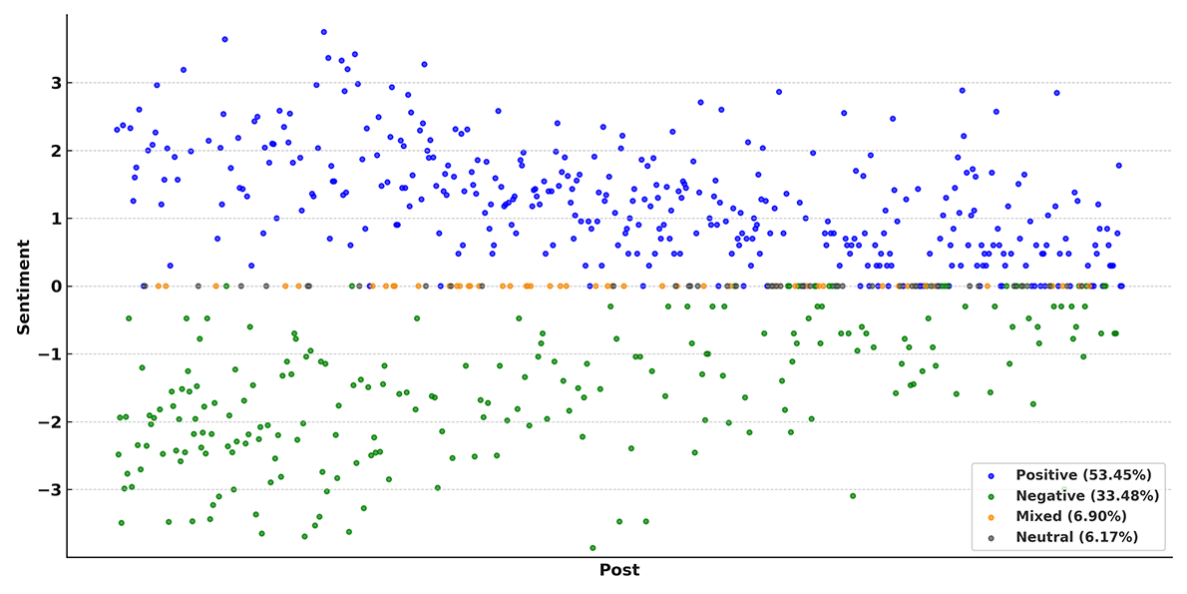
Sentiment distribution of posts on Xiaohongshu. The horizontal coordinate indicates the post, and the vertical coordinate indicates the sentiment score. Positive and negative posts were scored using their log-transformed like counts. Neutral and mixed posts were considered unclear in sentiment and scored as zero.

Sentiment analysis of the 681 posts under the usage interaction theme revealed that 53.45% (364/681) of the posts expressed positive sentiments, 33.48% (228/681) negative sentiments, 6.90% (47/681) mixed sentiments, and 6.17% (42/681) neutral sentiments (Figure 4). Positive posts frequently highlighted perceived benefits of e-cigarettes, such as the absence of tar and odor, with keywords like “satisfying” and “alternative.” Negative posts often focused on health concerns, addiction risks, or dissatisfaction with product quality, with common keywords including “harmful” and “deceptive.”

Notably, 6.90% (47/681) of the posts expressed mixed sentiments, acknowledging both advantages and drawbacks of e-cigarettes. Representative expressions included phrases like “tastes good but expensive” and “more addictive but still preferred,” highlighting a nuanced and conflicted user perspective.

### Engagement Metrics and Influencing Factors

Multivariate logistic regression analysis revealed several factors influencing user engagement (the number of comments). Of the 1729 posts analyzed, 1445 (83.5%) received at least 1 comment, while 284 (16.5%) received none. Variance inflation factor values were below 2, indicating no evidence of multicollinearity (Table S1 in Multimedia Appendix 1). Video posts were significantly more likely to generate comments than text-image posts, with an OR of 2.624 (95% CI 2.017‐3.439, *P*<.001). Postthematic classification also impacted engagement. Health hazards posts were 1.5 times more likely to elicit comments compared to advertising promotion posts (OR 1.498, 95% CI 1.099‐2.042, *P*=.01). Similarly, usage interaction posts had 1.46 times higher odds of generating comments than advertising promotion posts (OR 1.455, 95% CI 1.134‐1.869, *P*=.003). However, content length showed no significant association with the likelihood of receiving comments (OR 1.01, 95% CI 0.791‐1.293, *P*=.94). The results were shown in Table 3.

**Table 3.** The logistic multivariate analysis of user engagement metrics.

Factor		OR (95% CI)	*P* value
Post type			
	Text-image posts	Reference	—^a^
	Video posts	2.624 (2.017‐3.439)	<.001
Classification			
	Advertising promotion	Reference	—
	Health hazards	1.498 (1.099‐2.042)	.01
	Usage interaction	1.455 (1.134‐1.869)	.003
	Others	1.079 (0.758‐1.531)	.67
Content length			
	Long posts	Reference	—
	Short posts	1.01 (0.791‐1.293)	.94

a Not applicable.

## Discussion

### Principal Results

To the best of our knowledge, this is the first study in China to comprehensively analyze e-cigarette–related content on Xiaohongshu, a leading Chinese social media platform, over an extended period. The results indicate that e-cigarette contents are presented within multiple narratives, each playing a distinct role in shaping public perceptions.

First, e-cigarettes are prominently marketed as lifestyle products, with significant emphasis placed on visual branding, flavor diversity, and aesthetic appeal. Keywords such as “flavor,” “design,” and “fashion” frequently appear in promotional posts, reflecting a deliberate effort by marketers to associate e-cigarettes with modernity and trendiness. Similar to findings by Kreitzberg et al [21], this study observes that marketing strategies targeting young users often present e-cigarettes as symbols of sophistication and innovation, effectively normalizing their use [22,23]. However, our analysis reveals an additional trend where products are disguised as items like candy or milk tea cups, a tactic specifically aimed at lowering adolescents’ mental defenses. This targeted marketing is concerning, as studies by Berry and Sargent et al have demonstrated that adolescent e-cigarette use significantly increases the risk of transitioning to traditional smoking [24-26]. These findings emphasize the need for stricter regulatory oversight to curb such predatory practices.

Second, although health-related posts accounted for a relatively small proportion of the total content, they consistently received higher levels of user engagement, indicating their potential influence and public interest. However, their limited presence weakens their ability to counterbalance the dominant promotional narratives within the online discourse. A further challenge lies in the blurred boundaries between health information and marketing rhetoric. Some promotional posts present e-cigarettes as “harm reduction tools” or “safer alternatives to traditional cigarettes” [27], appropriating the language of public health to advance commercial agendas. This narrative strategy may dilute the impact of genuine health messaging and lead to a more complicated—or even misleading—public understanding of the actual risks associated with e-cigarette use [28]. However, we also recognize that engagement metrics such as likes, saves, and comments are based only on part users who have viewed posts. Due to privacy restrictions on the Xiaohongshu platform, the number of views is not public, which limits our ability to calculate accurate engagement rates. Therefore, the current metrics should be interpreted as a proxy for potential impact rather than a direct measure of reach.

Third, usage interaction dominates discussions, with significant focus on personal experiences and smoking cessation attempts. These posts reveal motivations such as quitting smoking and managing withdrawal symptoms, which are consistent with findings from Western studies that document e-cigarettes’ perceived role in harm reduction [27,29]. However, unlike these studies, our analysis highlights the dual perception of e-cigarettes in China: as both tools for harm reduction and fashionable consumer products. This duality is particularly problematic for younger users, who are more vulnerable to marketing tactics that downplay risks.

National regulations on e-cigarettes have influenced both the volume and direction of content on social media platforms. Since 2019, China has banned online sales and advertising of e-cigarettes; in 2021, e-cigarettes were officially classified as tobacco products, and in 2022, further regulations limited available flavors to tobacco and mandated product standardization [7,8]. These policies have significantly shaped the focus of online discussions. Following the release of the 2020 China Report on the health hazards of smoking in May 2021 [30], discussions related to nicotine, lung health, and secondhand smoke increased markedly, reflecting the influence of authoritative health information on public discourse. Beginning in 2023, there was a sharp rise in e-cigarette–related posts, likely driven by a combination of regulatory changes, intensified health communication, increased media coverage, and shifting public attitudes. Nonetheless, the rapid growth of Xiaohongshu’s user base also played a key role, providing greater momentum for content creation and dissemination.

Finally, the analysis of sentiment patterns reveals diverging opinions on e-cigarettes, with posts expressing both positive and negative sentiments. Positive narratives often emphasize convenience, reduced odor, and perceived harm reduction, framing e-cigarettes as superior to traditional smoking. Conversely, negative posts focus on addiction risks, health concerns, and dissatisfaction with product quality. In addition, some posts reflected ambivalent attitudes—acknowledging both benefits and harms—suggesting that public opinion is not strictly divided but rather complex and multifaceted. This range of sentiment illustrates how social media serves as a space where different narratives coexist and interact, shaped by personal experiences, promotional messaging, and evolving public awareness. We used like counts to score sentiment, but likes may not always reflect true emotions—especially for controversial posts. Neutral and mixed posts were scored as zero to reduce bias, though some emotional meaning may still be missed.

Together, these findings suggest that the regulation of the online e-cigarette market must be further strengthened. Although the Chinese government implemented a series of bans on e-cigarette sales, companies continue to exploit social media platforms like Xiaohongshu to circumvent these restrictions [1,31]. Stronger collaboration between regulatory bodies, social media platforms, and public health organizations is essential to address these issues. Platforms like Xiaohongshu must assume greater responsibility for monitoring and removing misleading or harmful content. Specifically, platforms should consider implementing automated detection systems to flag posts that contain health-related claims or promotional language associated with e-cigarettes; clearly labeling or warning users about commercial content that references health benefits; enhancing manual review processes for content that targets adolescents or uses deceptive forms (eg, products disguised as candy or beverages); and establishing formal channels for collaboration with public health authorities to review and remove posts that contradict scientific consensus or regulatory guidelines. Public health campaigns should focus on educating consumers about the risks associated with flavored e-cigarettes and unregulated products, countering the pervasive narrative of e-cigarettes as harmless or beneficial alternatives. Together, these measures can help create a safer and more transparent e-cigarette market while mitigating the health risks posed by these products.

### Limitations

This study has several limitations. Data were collected from social media, and the user groups of these platforms are predominantly younger users who are familiar with the internet, resulting in an underrepresentation of older age groups who do not frequently use social media in the sample. In addition, although we used a time-stratified random sampling strategy to improve temporal coverage, the resulting dataset may not represent all e-cigarette–related discourse on Xiaohongshu. Nonetheless, given that the main consumer group of e-cigarettes is young people, our study is still a good reflection of the general attitudes of the target population. To more accurately assess consumer attitudes, future research should include surveys across various social media platforms as well as traditional media. Another limitation concerns the measurement of user engagement. Metrics such as likes, comments, and saves reflect only active interactions and do not account for passive exposure. Since Xiaohongshu does not make view counts publicly available, we were unable to calculate more objective engagement rates based on content visibility. This limitation may introduce bias in evaluating actual user engagement and the influence of posts. In addition, due to data access restrictions on the platform, our analysis was limited to a small set of publicly available postlevel variables. Unobserved confounders—such as user account characteristics or algorithmic exposure—may have influenced engagement outcomes. Although the sentiment labeling followed a rigorous protocol with adjudication by a third researcher, the absence of inter-rater reliability metrics remains a methodological limitation and should be addressed in future studies through formal validation and reliability testing. This study initially used the Latent Dilettante Assignment topic model; however, despite several adjustments to the number of topic words, the model still struggled to achieve clear topic differentiation. This limitation may be due to the fact that the Latent Dilettante Assignment model relies on bag-of-words representations, which are unable to capture the semantic relationships between words and do not accurately reflect the true content of a document [32]. We therefore used NVivo software for content coding and qualitative analysis, which may be affected by coder bias.

### Conclusions

This study highlights the complex interplay between promotional content, health messaging, and user narratives surrounding e-cigarettes on Xiaohongshu. While promotional posts dominate, the high engagement with health-related content reflects a demand for accurate information. The persistence of misleading marketing tactics underscores the need for stricter regulation and enhanced oversight.

Strengthened collaboration between policy makers, social media platforms, and public health organizations is essential to ensure compliance and counter misinformation. Leveraging social media for health education presents a critical opportunity to protect vulnerable groups and foster informed decision-making. These findings provide a foundation for effective public health strategies and policy development in addressing e-cigarette use.

## Supplementary material

10.2196/71173 Multimedia Appendix 1 Supplementary table 1: regression model VIF. VIF: variance inflation factor.

## Abbreviations

e-cigarette: electronic cigarette
OR: odds ratio
